# Transcatheter tricuspid valve intervention versus optimal medical therapy alone for severe tricuspid regurgitation: an updated meta-analysis with reconstructed time-to-event data

**DOI:** 10.1016/j.ijcha.2025.101794

**Published:** 2025-09-13

**Authors:** Ahmed Ibrahim, Laila Shalabi, Sofian Zreigh, Shrouk Ramadan, Ahmed Samir, Mohamed Adel Elsawy, Mohamed Mahmoud Fathy, Belal Mohamed Hamed, Hossam Elbenawi, Mustafa Turkmani, Ahmed Y. Azzam, Hani Mahmoud-Elsayed, Islam Y. Elgendy

**Affiliations:** a Faculty of Medicine, Alexandria University, Alexandria, Egypt; b Faculty of Medicine, Gharyan University, Gharyan, Libya; c Faculty of Medicine, Ankara Yıldırım Beyazıt University, Ankara, Turkey; d Faculty of Medicine, Ain Shams University, Cairo, Egypt; e Faculty of Medicine, Minia University, Minya, Egypt; f Faculty of Medicine, Al-Azhar University, Cairo, Egypt; g Faculty of Medicine, Al-Azhar University, New Damietta, Egypt; h Department of Cardiovascular Medicine, Mayo Clinic, Rochester, USA; i Division of Pulmonary and Critical Care, University of Toledo, Toledo, OH, USA; j Faculty of Medicine, Michigan State University, East Lansing, MI, USA; k Medical Big Data Research Center, SNU Medical Research Center, Seoul National University (SNU), Seoul, South Korea; l Department of Cardiology, Aswan Heart Center, Magdi Yacoub Foundation, Aswan, Egypt; m Division of Cardiovascular Medicine, Gill Heart Institute, University of Kentucky, Lexington, KY, USA

**Keywords:** Transcatheter tricuspid valve intervention, Optimal medical therapy, Severe tricuspid regurgitation, All-cause mortality, Heart failure hospitalization, Meta-analysis

## Abstract

**Background:**

Severe tricuspid regurgitation (TR) is strongly associated with high mortality and morbidity. This *meta*-analysis aims to compare the outcomes of transcatheter tricuspid valve intervention (TTVI) versus optimal medical treatment (OMT) alone among patients with severe TR.

**Methods:**

Electronic databases were systematically searched to identify randomized controlled trials (RCTs) and propensity score-matched observational studies comparing TTVI with OMT. The primary outcome was all-cause mortality. Summary estimates were calculated using a random-effects model.

**Results:**

Five studies (3 RCTs, 2 observational; *n* = 1988 patients) were included. TTVI was associated with a nonsignificant trend toward a lower incidence of all-cause mortality (risk ratio [RR]: 0.70, 95 % confidence interval [CI] 0.48–1.03; P = 0.071), primarily driven by observational studies. TTVI demonstrated significant benefits in terms of TR severity reduction (RR: 7.82, 95 % CI 5.60–10.93; P < 0.0001), enhanced health status as measured by the Kansas City Cardiomyopathy Questionnaire (mean difference: +14.46 points, 95 % CI 11.55–17.38; p < 0.0001), and reduced heart failure (HF) hospitalization rates (RR: 0.73, 95 % CI 0.56–0.96; P = 0.025). However, TTVI was associated with an increased risk of major bleeding (RR: 3.21, 95 % CI 1.61–6.39; P = 0.0009).

**Conclusion:**

Among patients with severe TR, TTVI was not statistically associated with a lower incidence of all-cause mortality but was associated with a reduced risk of HF hospitalization, significant reduction in TR severity, and improved quality of life. Future large RCTs with extended follow-up are needed to confirm these findings and identify the subset of patients who benefit the most.

**Systematic review protocol:** CRD420251002402 (PROSPERO)

## 1 Introduction

Tricuspid regurgitation (TR) is a frequent valvular disorder that has different etiologies. Moderate or severe TR affects approximately 0.5 % of adults, with prevalence rising to nearly 4 % in those over 75 years old [1]. Without treatment, TR can lead to progressive right heart failure (HF) [2]. Although medical management (e.g., diuretics, HF medications) can offer symptomatic relief from volume overload, it remains mainly palliative [3]. This approach fails to address the underlying valvular pathology, ultimately leading to progressive right ventricular dysfunction. Long-term studies highlight the poor prognosis of severe TR with medical management alone, with two-year mortality nearing 50 % [4]. While surgical intervention is also an option, only a few patients are considered surgical candidates [5], and isolated surgical repair/replacement is associated with significant mortality. Accordingly, most patients with severe TR are managed mainly conservatively.

In recent years, transcatheter tricuspid valve interventions (TTVI) have emerged as a less invasive alternative for high-risk patients. These include tricuspid edge-to-edge repair (TEER), annuloplasty devices (Cardioband), and transcatheter tricuspid valve replacement (TTVR) devices (EVOQUE, LuX-Valve) [6]. Early observational studies of TTVI reported promising signals of improved survival in addition to reduction in TR severity and enhancements in quality of life [[7], [8], [9]].

These findings may be promising, however, clinical guidelines continue to prioritize medical therapy as the first-line approach and remain cautious about TTVI due to limited randomized evidence. The 2021 European Society of Cardiology (ESC) and the European Association for Cardio-Thoracic Surgery (EACTS) guidelines assign a Class IIb recommendation to TTVI, suggesting it may be considered for patients with severe symptomatic TR who are inoperable or at prohibitive surgical risk, while highlighting the lack of robust RCTs [10]. Similarly, the 2020 American College of Cardiology (ACC) and the American Heart Association (AHA) guidelines recommend medical therapy as the primary approach for TR [11]. While acknowledging the emerging role of transcatheter interventions, they refrain from broad endorsement, emphasizing the ongoing need for more conclusive evidence. Both guidelines underscore the need for further data before broader clinical adoption of transcatheter therapies.

This call for high quality evidence has been met by recent randomized controlled trials (RCTs).While these trials confirmed that TTVI leads to a significant reduction in TR severity and an improvement in disease-specific health status, they demonstrated no statistically significant difference in hard clinical outcomes, including all-cause mortality and HF hospitalization, potentially due to limited statistical power [12,13].

Hence, we performed this *meta*-analysis to synthesize available data comparing transcatheter interventions with medical treatment, aiming to comprehensively evaluate the efficacy and safety of transcatheter interventions.

## 2 Methods

### 2.1 Protocol and registration

This systematic review was conducted in accordance with Cochrane standards [14] and the Preferred Reporting Items for Systematic Reviews and Meta-Analyses (PRISMA) guidelines [15]. The protocol was registered with PROSPERO (registration number: CRD420251002402) [16].

### 2.2 Search strategy

A comprehensive literature search was performed across multiple databases, including PubMed, Scopus, Cochrane, and Web of Science, up to Aug 10, 2025, without any language restrictions. The search utilized MeSH terms pertinent to (transcatheter tricuspid valve interventions, encompassing medical treatment, replacement, and repair). Additionally, backward snowballing was used to identify additional studies through a review of references and related articles. The detailed search strategy for each database is provided in Table S1.

### 2.3 Selection criteria

To minimize the risk of selection bias, we included only RCTs and propensity score-matched (PSM) observational studies that compared TTVI (repair or replacement) with optimal medical treatment (OMT) in adult patients with at least moderate-to-severe TR. The pre-specified clinical outcomes of interest included all-cause mortality, heart failure hospitalization, change in Kansas City Cardiomyopathy Questionnaire (KCCQ) score, change in 6-minute walk distance (6MWD), reduction in tricuspid regurgitation severity, major bleeding, cardiovascular mortality, HF-related mortality, and stroke.

### 2.4 Study selection process

After removing duplicates using Mendeley, studies were imported into Rayyan. Where two reviewers (A.S and M.M) independently screened titles and abstracts, and conflicts were resolved by a third reviewer (L.S). Eligible studies were then assessed in full-text by two reviewers (L.S and A.I) and any conflicts were resolved through discussion.

### 2.5 Data extraction

Four reviewers (A.S, M.M, S.Z and M.E) independently extracted data using a standardized sheet, which included the following information: study characteristics (first author's last name, registry number, recruitment period, inclusion and exclusion criteria, patient characteristics, and comorbidities), as well as outcomes of interest. The extracted data were subsequently reviewed by a fifth reviewer (A.I) for accuracy.

### 2.6 Quality assessment

The quality of included RCTs was assessed using the Cochrane Risk of Bias Tool (RoB 2), which evaluates the risk of bias across five domains, including randomization, deviation from intended interventions, missing outcome data, measurement of the outcome, and selection of the reported result [17]. For non-randomized studies, the ROBINS-I tool was used to evaluate seven domains: confounding bias, selection bias, classification bias, deviations from intended interventions, missing data bias, measurement bias, and the selection of reported results [18]. Each study was assessed by two independent reviewers (A.S, M.M., S.Z, and M.E) and any conflicts were resolved through discussion with (L.S).

### 2.7 Statistical analysis

The statistical analysis was performed using R version 4.4.2 (R Foundation for Statistical Computing) with the “meta” package. A random-effects model was used for all analyses due to anticipated clinical and methodological heterogeneity [19]. Risk ratios (RR) with 95 % confidence intervals (CI) were calculated to measure effect sizes for dichotomous outcomes [20], while mean differences (MD) with 95 % CI were used for continuous outcomes. A two-sided P-value of < 0.05 was considered statistically significant. Heterogeneity among studies was assessed using the I2 statistic, following Cochrane's guidelines. Specifically, an I2 value of 0 % to 40 % indicates minimal heterogeneity, 30 % to 60 % suggests moderate heterogeneity, 50 % to 90 % reflects substantial heterogeneity, and values between 75 % and 100 % signify considerable heterogeneity [21]. Subgroup analyses were conducted based on the types of included studies (RCTs versus observational studies) and intervention strategy (repair vs replacement). Due to the limited number of studies, we did not assess publication bias [22].

To reconstruct individual patient data (IPD) for all-cause mortality and HF hospitalization, we employed a methodology described by Liu et al. [23]. This approach was used to overcome aggregate data limitations, enabling a more detailed analysis with precise hazard ratio estimation and accounting for censoring and varying follow-up times. We used WebPlotDigitizer to extract survival probabilities from Kaplan-Meier curves and then combined these with patient-at-risk data to estimate IPD using the 'IPDfromKM' package in R. To ensure accuracy, we compared our reconstructed data with the original studies' Kaplan-Meier curves and “total at risk” tables. We accepted results with a root mean square error ≤ 0.05, a mean absolute error ≤ 0.02, and a max absolute error ≤ 0.05. We used the Mann-Whitney test and Bootstrap *t*-test to compare the distributions of the original and reconstructed survival curves. Cox proportional hazards models were used to calculate overall hazard ratios (HR) and 95 % confidence intervals (CI) from the reconstructed IPD.

## 3 Results

### 3.1 Literature search results

**Fig. 1:**
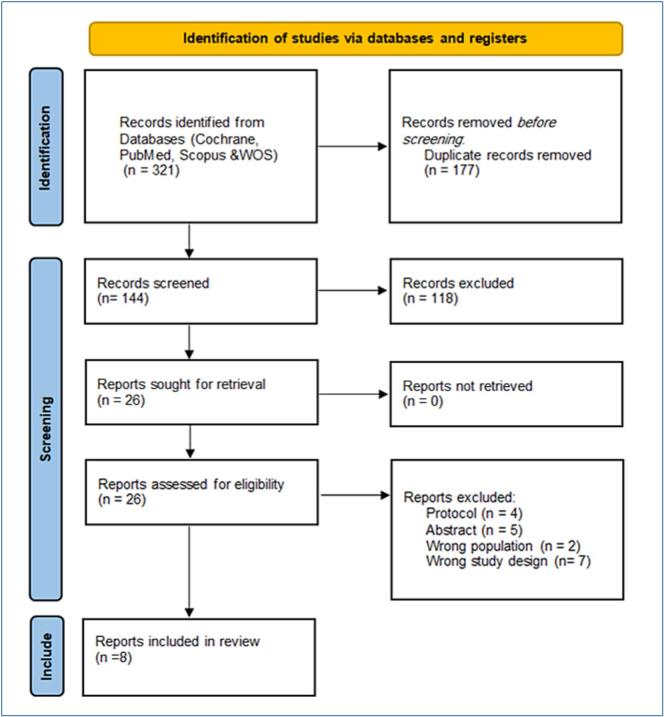
PRISMA flowchart of study selection process.

A total of 320 articles were initially identified through the search process. After removing duplicates, 144 unique articles remained. Following a title and abstract screening, 118 articles were excluded, leaving 26 studies for full-text screening. Ultimately, 5 studies (represented in 8 records) met the inclusion criteria and were included in the final analysis [8,12,13,[24], [25], [26], [27], [28]]. The final analysis included 2 observational studies and 6 published records from 3 RCTs: 3 records from the TRILUMINATE trial, including one with a 2-year follow-up [25] and two reporting distinct 1-year outcomes [12,27], as well as two records from the TRISCEND II trial, focusing on safety and efficacy outcomes [13] and quality of life outcomes [28], respectively, and one record from the Tri.FR trial [24]. A summary of the study selection process is provided in the PRISMA flowchart **(**Fig. 1**)**.

### 3.2 Characteristics of included studies

The pooled analysis included 1,988 patients (1058 in the TTVI group and 930 in the OMT group). The follow-up duration was mostly up to one year, with one study extending to 2 years. Notably, three of the included studies involved only patients with severe TR, while the TRILUMINATE trial included a small proportion (2.2 % in the TEER group and 1.5 % in the OMT group) of patients with moderate TR. Additionally, the study by Kresoja et al. involved 4.7 % of patients with moderate to severe TR. Kresoja et al., TRILUMINATE trial, Tri.FR trial primarily assessed the efficacy of TEER [12,24,26], while TRISCEND II focused on the clinical outcomes of TTVR [13]. Notably, Taramasso et al adopted a comprehensive approach, investigating both repair and replacement techniques [8]. The key characteristics of the studies included in our analysis are presented in Table 1, Table 2**.**

**Table 1:** Main characteristics of the included study.

Study ID	Country	Study Design	Registry number	Recruitment period	Intervention	Control	Total sample size	Follow up	Severity of Tricuspid regurgitation	Device	Main inclusion criteria
Arnold et al. 2025 (TRISCEND II)	United States and Germany	Multinational RCT	NCT04482062	May 2021 through April 2023	Transcatheter tricuspid-valve replacement	OMT	392	1-year	At least severe TR	EVOQUE system	1. Severe TR (grade 3–5)2. TR symptoms/hospitalization for HF3. Eligible for EVOQUE valve replacement
Hahn et al. 2025 (TRISCEND II)											
Donal et al. 2025 (Tri.Fr)	France and Belgium	Multicenter RCT	NCT04646811	March 2021 − March 2023 (last follow-up April 2024)	Transcatheter Tricuspid Valve Edge-to-Edge Repair	OMT	300	1-year	At least severe TR	TriClip System.	3. HF signs in past 12 months4. Stable, optimized medical treatment5. Ineligible for surgical correction (by heart team)
Kresoja et al. 2020	Germany	PSM retrospective cohort study	−	July 2016 – April 2019	Leaflet transcatheter edge-to-edge repair technique	OMT	188	1-year	At least moderate to severe TR	MitraClip	1. Symptomatic, moderate to severe TR 2. NYHA functional class II-IV 3. HF symptoms despite OMT4. High or prohibitive surgical risk5. Suitable tricuspid anatomy for edge-to-edge repair
Sorajja et al. 2023 (TRILUMINATE)	USA	RCT	NCT03904147	August 21, 2019- September 29, 2021	Transcatheter Tricuspid Valve Repair system.	Optimal OMTfor at least 30 days before randomization	350	1-year	Severe TR	TriClip system	1. Severe TR (confirmed by echo lab)2. Symptomatic (NYHA II-IVa) 3. PASP < 70 mmHg 4. Stable HF medical therapy ≥ 30 days5. No other cardiovascular conditions requiring intervention6. Intermediate or greater surgical risk
Tang et al. 2024 (TRILUMINATE)				August 21, 2019 − June 29, 2022			572				
Kar et al. 2025 (TRILUMINATE)								2-year			
Taramasso et al. 2019	Switzerland	PSM retrospective cohort study	−	2016 to 2018	Transcatheter tricuspid valve intervention	OMT	536	11 (IQR: 4 to 28) months	At least severe TR	MitraClip, FORMA, Cardioband, TriCinch, TriAlign, PASCAL, NaviGate, and caval valve devices	1. Severe/greater symptomatic TR (per EU/US guidelines)2. Intervention decision by multidisciplinary team after clinical/anatomical assessment.

**Abbreviations:** OMT: Optimal Medical Treatment, T-TEER: Transcatheter Tricuspid Valve Edge-to-Edge Repair, TR: Tricuspid Regurgitation, TVR: Tricuspid Valve Replacement, NYHA: New York Heart Association, TEER: Transcatheter Edge-to-Edge Repair, HF: Heart Failure, PASP: Pulmonary Artery Systolic Pressure, RCT: Randomized Controlled Trial, PSM: Propensity Score Matching.

**Table 2:** Baseline characteristics of patients of the included studies.

Study ID	Sample size		Age (mean-SD)		Male, n(%)		BMI (Kg/m2)		Atrial fibrillation, n(%)		CKD, n(%)		Hypertension, n(%)		Functional tricuspid regurgitation, n(%)		NYHA class ≥ 3, n(%)		Baseline medications, n (%	
	TTVI	OMT	TTVI	OMT	TTVI	OMT	TTVI	OMT	TTVI	OMT	TTVI	OMT	TTVI	OMT	TTVI	OMT	TTVI	OMT	TTVI	OMT
Arnold et al. 2025	259	133	79.3 ± 1.34	79.1 ± 1.94	65 (25.1)	31(23.3)	26.8 (26.1–27.6)*	27.0 (26.1–27.9)*	249 (96.1)	123 (92.5)	140 (54.1)	79 (59.4)	235 (90.7)	122 (91.7)	192 (74.1)	95 (71.4)	189 (73.0)	92 (69.2)	−	−
Hahn et al. 2025																				
Donal et al. 2025	152	148	78.3 ± 6.4	78.7 ± 6.4	54 (34.5)	55 (37.2)	25.2 (22.4–28.7)*	25.0 (22.8–28.9)*	143 (94.1)	143 (94.1)	13 (8.55)	6 (4.05)	106 (69.7)	102 (68.9)	−	−	59 (38.9)	68 (45.9)	Beta-blockers: 107 (70.4) Loop diuretic: 145 (95.4)	Beta-blockers: 110 (74.3) Loop diuretic: 143 (96.6)
Kresoja et al. 2020 (HFpEF)	61	61	77.3 ± 5.62	77.83 ± 7.21	29 (47.5)	22 (36)	25.2 (23.0–30.3)*	25.1 (22.0–27.8)*	55 (92)	48 (79)	2(3)	6(10)	−	−	61(100)	61(100)	52 (85)	38 (64)	Beta-blockers: 54 (89) ACEi/ARB: 44 (72) Diuretics: 56 (93)	Beta-blockers: 36/44 (82) ACEi/ARB: 35/44 (77) Diuretics:37/44 (84)
Kresoja et al. 2020 (HFrEF)	33	33	76.87 ± 6.51	75.67 ± 11.62	17 (51.5)	18 (54.5)	26.0 (23.5–29.9)*	25.8 (22–8–26.2)*	27 (82)	23 (70)	4(12)	3(9)	−	−	33(100)	33(100)	28 (85)	28 (85)	Beta-blockers: 29 (91) ACEi/ARB: 26 (81) Diuretics: 97 (31)	Beta-blockers: 21/22 (96) ACEi/ARB: 19/22 (86) Diuretics: 21/22 (96)
Sorajja et al. 2023	175	175	78.0 ± 7.4	77.8 ± 7.2	77 (44)	81 (46.3)	27.0 ± 5.8	26.9 ± 5.2	153 (87.4)	162 (92.6)	62 (35.4)	62 (35.4)	142 (81.1)	141 (80.6)	165 (94.8)	158 (92.9)	104 (59.4)	97 (55.4)	Beta-blockers: 114 (65.1) ACEi, ARB, or ARNI: 68 (38.9) Diuretic: 152 (86.9)	Beta-blockers: 115 (65.7) ACEi, ARB, or ARNI:66 (37.7) Diuretic: 161 (92.0)
Tang et al. 2024	285	287	78.1 ± 7.9	78.1 ± 7.6	125 (43.8)	132 (45.9)	26.8 ± 5.8	27.1 ± 5.5	236 (82.8)	266 (92.7)	91 (31.9)	100 (34.8)	231 (81.1)	234 (81.5)	270 (95.7)	263 (93.9)	160 (56.1)	155 (54)	Beta-blockers: 198 (69.5) ACEi: 39 (13.7) ARBs: 77 (27) Diuretics: 274 (96.1)	Beta-blockers: 208 (72.5) ACEi: 36 (12.5) ARBs: 94 (32.8) Diuretics: 282 (98.3)
Kar et al. 2025																				
Taramasso et al. 2019	268	268	76 ± 13	78 ± 8	118 (44)	110 (41)	−	−	220 (82)	134 (50)	−	−	91 (34)	78 (29)	241 (90)	256 (95)	249 (93)	62 (23)	−	−

*Median and interquartile range.

**Abbreviations: BMI:** Body Mass Index, **CKD:** Chronic Kidney Disease, **HFpEF:** Heart Failure with preserved Ejection Fraction, **HFrEF:** Heart Failure with reduced Ejection Fraction, **NYHA:** New York Heart Association, **OMT:** Optimal Medical Treatment, **TTVI:** Transcatheter Tricuspid Valve Interventions, **ACEi:** Angiotensin-Converting Enzyme inhibitors, **ARB:** Angiotensin Receptor Blockers, **ARNI:** Angiotensin Receptor-Neprilysin Inhibitors.

### 3.3 Risk of bias

TRISCEND II and TRILUMINATE trials had an overall low risk of bias, despite being open-label; assessments were blinded. Tri.Fr trial had overall some concerns (Fig. S1). On the other hand, the 2 observational studies were found to have a moderate risk of bias. The main risks observed were deviations from the intended intervention, because none of the research provided detailed data on the interventions, and selective reporting, as none of the studies had a published protocol (Fig. S2).

### 3.4 Meta-analysis of primary outcome (All-cause mortality)

**Fig. 2:**
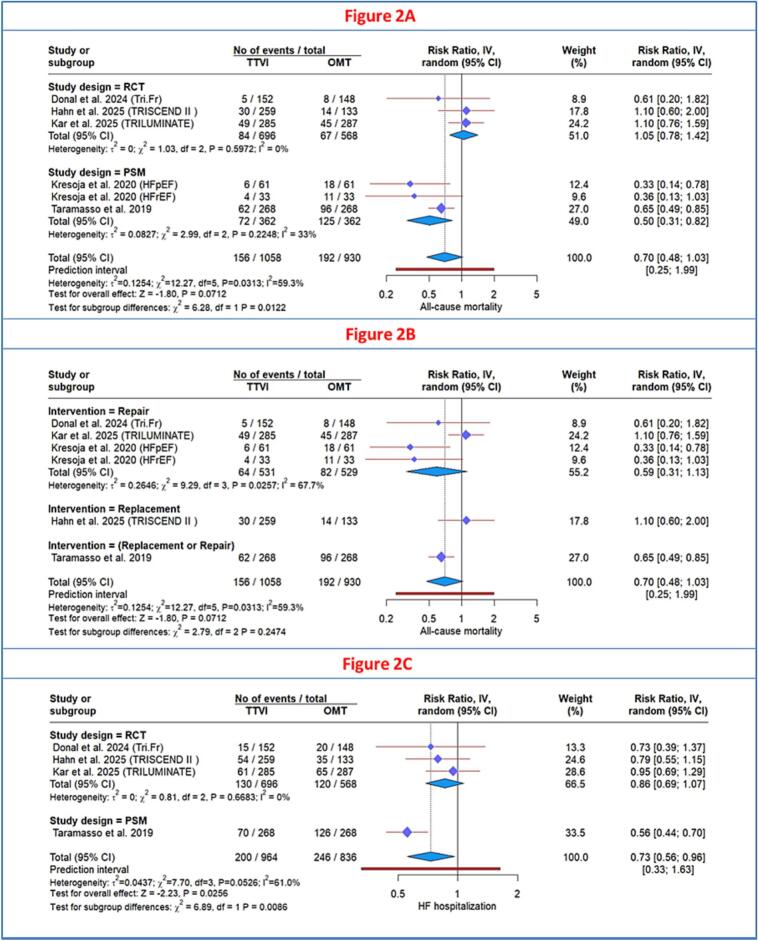
**A:** Forest plot of all-cause mortality (subgroup analysis by study design). B: Forest plot of all-cause mortality (subgroup analysis by intervention strategy). C: Forest plot of heart failure hospitalization (subgroup analysis by study design).

Three RCTs [13,24,25] and 2 observational studies [8,26] assessed all-cause mortality between TTVI and OMT groups. There was no statistically significant difference between the two groups (RR: 0.70; 95 % CI, 0.48–1.03; P = 0.071) with moderate heterogeneity (I^2^ = 59.3 %) (Fig. 2A**)**. To resolve heterogeneity, subgroup analysis by study design was performed and revealed significant subgroup differences (P = 0.01): observational studies favored TTVI (RR: 0.50, 95 % CI 0.31–0.82; I^2^ = 33 %), whereas RCTs demonstrated no significant benefit (RR: 1.05; 95 % CI, 0.78–1.42) with no heterogeneity (I^2^ = 0 %). However, no significant difference was observed between TTVI techniques, repair versus replacement (p = 0.24) (Fig. 2B**)**.

**Fig. 3:**
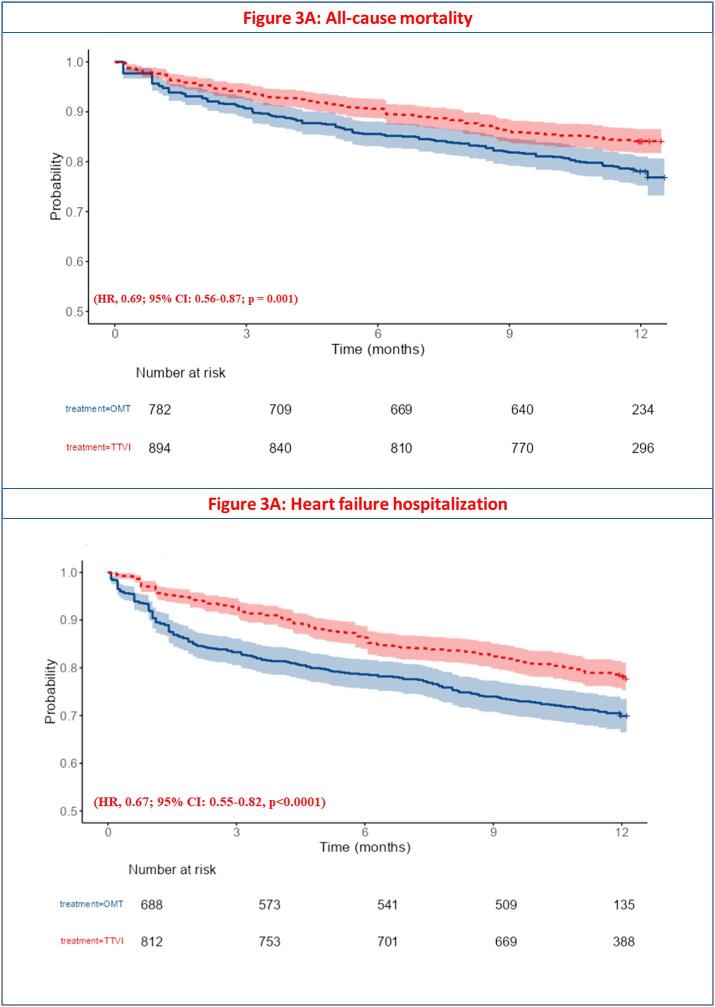
**A:** Kaplan-Meier survival analysis for all-cause mortality. B: Kaplan-Meier survival analysis for heart failure hospitalization.

Reconstruction of Kaplan-Meier survival curves from four studies [8,12,13,26] revealed that TTVI was associated with a significant 1-year survival advantage over OMT, characterized by a 6 % absolute risk reduction (TTVI: 84.1 % vs. OMT: 78.1 %) and relative reduction in mortality risk (HR, 0.69; 95 % CI: 0.56–0.87; p = 0.001) (Fig. 3A**)**. A subgroup analysis by study design yielded disparate findings: RCTs [12,13] showed no significant difference (HR: 1.22; 95 % CI: 0.83–1.79; p = 0.32) (Fig. S3), whereas observational studies [8,26] demonstrated a significantly lower mortality incidence with TTVI (HR: 0.54, 95 % CI: 0.41–0.72; p < 0.001) **(**Fig. S4).

### 3.5 Meta-analysis of secondary outcomes

#### 3.5.1 Heart failure hospitalization

Four studies [8,13,24,25] evaluated HF hospitalization with 964 patients in the TTVI group and 836 patients in the OMT group. TTVI was associated with a lower rate of HF hospitalization (RR, 0.73; 95 % CI, 0.56–0.96; *P* = 0.025) with substantial heterogeneity (I^2^ = 61 %) (Fig. 2C). To resolve heterogeneity, subgroup analysis by study design was performed and revealed significant subgroup differences (P = 0.0086): observational studies favored TTVI (RR: 0.56, 95 % CI 0.44–0.70), whereas RCTs demonstrated no significant benefit (RR: 0.86; 95 % CI, 0.69–1.07) with no heterogeneity (I^2^ = 0 %).

Furthermore, two trials [13,25] evaluated annualized HF hospitalization rates. At 1 year, the TTVI and OMT cohorts showed comparable rates (TRISCEND II: 9.7 % vs. 10.0 %; TRILUMINATE: 0.17 vs. 0.20 events/patient-year, P = 0.40). However, extended follow-up to 2 years in TRILUMINATE revealed a significant reduction in recurrent HF hospitalization with TTVI (0.19 vs. 0.26 events/patient-year, P = 0.02) [25].

Kaplan-Meier survival analyses derived from three included studies [8,12,13] demonstrated a significant reduction in 1-year HF hospitalization risk in the TTVI group compared to the OMT group (HR, 0.67; 95 % CI: 0.55–0.82, p < 0.0001) (Fig. 3B**)**.

#### 3.5.2 Change in KCCQ score from baseline

**Fig. 4:**
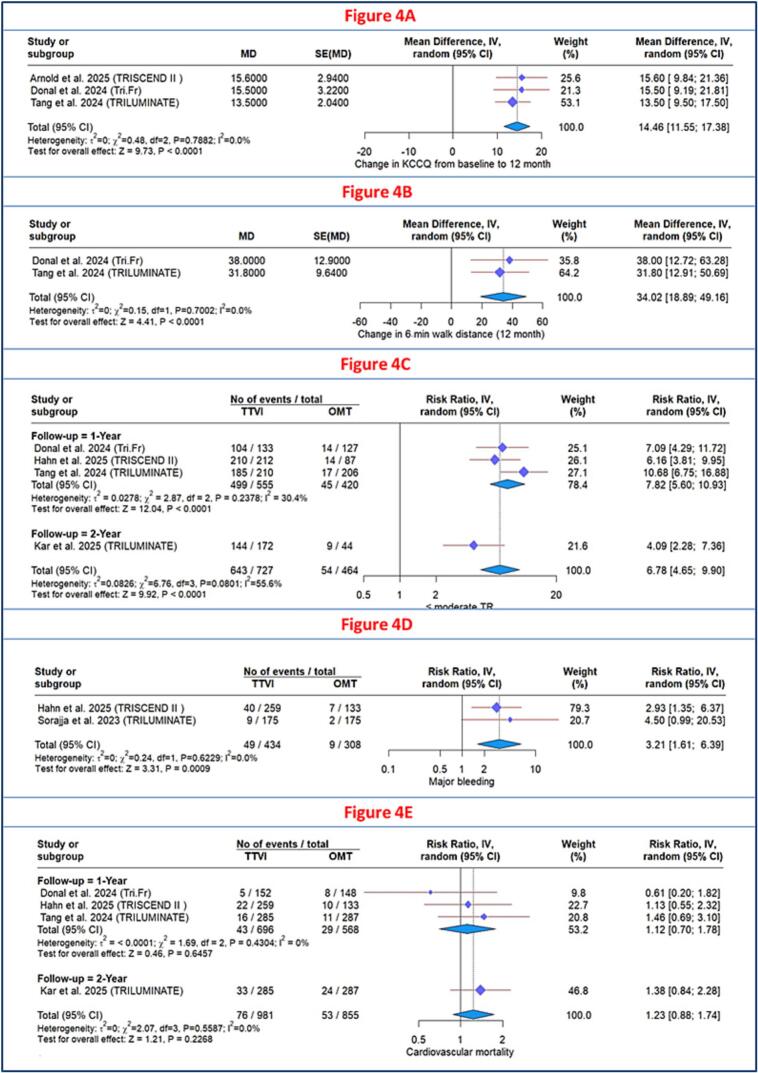
**A:** Forest plot of change in KCCQ score. B: Forest plot of change in 6-minute walk distance. C: Forest plot of tricuspid regurgitation severity reduction to ≤moderate. D: Forest plot of major bleeding risk. e: forest plot of cardiovascular mortality.

Three RCTs [12,24,28] evaluated the change in KCCQ score. The overall analysis demonstrated a significant improvement in health status favoring TTVI over OMT (MD: 14.46; 95 % CI, 11.55 to 17.38; P < 0.0001), with no heterogeneity (I^2^ = 0.0 %) (Fig. 4A).

#### 3.5.3 Change in 6-minute walk distance

Two RCTs [12,24] evaluated the change in 6MWD at 12-month follow-up. The pooled analysis demonstrated a statistically significant improvement in the TTVI group compared to the OMT group (MD: 34.02 m; 95 % CI, 18.89 to 49.16; P < 0.0001), with no heterogeneity (I^2^ = 0.0 %) (Fig. 4B).

#### 3.5.4 Reduction in TR severity

Three RCTs [12,13,24,25] assessed the efficacy of TTVI in reducing TR severity. The overall analysis demonstrated that TTVI significantly reduced TR severity, with a higher proportion of patients achieving ≤ moderate TR compared to OMT at one year (RR: 7.82; 95 % CI 5.60–10.93; P < 0.0001), with mild heterogeneity (I^2^ = 30.4 %). This benefit was sustained through two-year follow-up (RR: 4.09; 95 % CI 2.28–7.36; P < 0.001) (Fig. 4C).

#### 3.5.5 Major bleeding

Two RCTs [13,27] assessed major bleeding at 1 year, involving 434 patients in the TTVI group and 308 patients in the OMT group. TTVI was associated with a higher incidence of major bleeding (RR: 3.21; 95 % CI, 1.61–6.39; P = 0.0009), with no heterogeneity (I^2^ = 0.0 %) (Fig. 4D).

#### 3.5.6 Cardiovascular mortality

Three RCTs [12,13,24,25] assessed cardiovascular mortality. The analysis showed no significant difference between the two groups at either 1 year (Risk Ratio (RR: 1.12, 95 % CI 0.70–1.78, P = 0.65, I^2^ = 0.0 %) or 2 years (RR: 1.38, 95 % CI 0.84–2.28) (Fig. 4E).

#### 3.5.7 Heart failure-related mortality

Two RCTs [12,13,25] assessed HF-related mortality. The analysis showed no significant difference between the two groups at either 1 year (RR: 1.29, 95 % CI 0.66–2.53, P = 0.46, I^2^ = 0.0 %) or 2 years (RR: 1.29, 95 % CI 0.71–2.33) **(**Fig. S5).

#### 3.5.8 Stroke

Three RCTs [12,13,24] assessed stroke with 696 patients in the TVI group and 568 patients in the OMT group. The results showed no significant difference between the two groups (RR: 1.55; 95 % CI, 0.46–5.17; P = 0.48), with no heterogeneity (I^2^ = 0.0 %) (Fig. S6).

## 4 Discussion

In this updated *meta*-analysis of 5 studies involving 1988 patients, primarily those with severe TR, we demonstrated that TTVI was associated with a significant reduction in TR severity, an improvement in quality of life, and a lower rate of HF hospitalization, a finding driven by observational studies. TTVI was not associated with a lower incidence of mortality, but a higher incidence of major bleeding. These findings support the potential role for TTVI among select patients with severe TR, in which no other therapies are available.

In our analysis, TTVI yielded durable reductions in TR severity, with a significant proportion (80 %) of patients achieving ≤ moderate TR at 2 years, thereby demonstrating superiority over OMT. This anatomical improvement translated into clinically meaningful enhancements in both quality of life and objective functional capacity. The therapeutic benefits were evidenced by substantial improvements in KCCQ scores, reflecting reduced symptoms of fatigue, fluid retention, and physical limitations. Furthermore, patients demonstrated a significant increase in 6MWD (MD: 34 m), providing objective evidence of improved exercise tolerance and functional status. This robust improvement in functional capacity underscores how the reduction in TR severity translates into tangible gains in patients' physical performance and daily activities. These findings are consistent with the results of the TRISCEND II study, which demonstrated that over 95 % of patients undergoing TTVR achieved significant TR reduction within the first year [29]. Similarly, the TRILUMINATE trial reported that over 85 % of patients achieved ≤ moderate TR at 1-year follow-up with TEER therapy [12,25].

Moreover, our findings revealed that TTVI was not significantly associated with a lower incidence of all-cause mortality. This aligns with recent RCTs, which found no survival advantage at 1- or 2-year follow-up despite significant TR reduction [13,25]. The average left ventricular ejection fraction (LVEF) persists as a notably important prognostic and modifying feature in the context of tricuspid regurgitation and was poorly assessed among the studies. Lower LVEF is associated with worsening clinical outcomes [30] and, in many cases, diminishes the advantages of structural procedures, such as TTVI. The absence of stratification or subgrouping by LV systolic function is a limitation in drawing strong conclusions about LV function's impact on TTVI outcomes. Future studies should focus on clearly defining LV function to accurately categorize patients expected to benefit clinically. Even though reconstructed survival analysis indicated a favorable trend in 1-year mortality with TTVI compared to OMT, this observation was primarily influenced by observational data, even after using propensity score matching. Such non-randomized studies retain the possibility of unexplained biases due to confounding variables, which weakens the certainty of mortality conclusions. As a result, the mortality findings should be interpreted with caution.

The association of TTVI with a lower incidence of HF hospitalization was primarily derived from observational studies, as the pooled analysis of RCTs showed comparable results with only a trend favoring TTVI. Nevertheless, a statistically significant reduction in annualized HF hospitalization rates was evident at 2 years, suggesting a delayed therapeutic benefit of TTVI that is detectable only upon longer-term follow-up and underscoring the importance of accounting for repeated events in the analysis of clinical outcomes. These findings are corroborated by the TRILUMINATE trial [25], which demonstrated that despite progressive deterioration of TR and quality of life during the initial 12 months of follow-up, no significant increase in HF hospitalization was observed. Notably, approximately 50 % of patients exhibited a pronounced increase in annualized HF hospitalization rates from 0.17 to 0.50 events/patient-year during the second year of follow-up, which subsequently decreased to 0.35 events/patient-year following crossover to TTVI, thereby underscoring the sustained clinical efficacy of TTVI over time.

Given the TR burden, TTVI has evolved as a therapeutic strategy, particularly for HF patients for whom surgical repair is not an option. This wide range of therapy alternatives (valve repair and replacement operations) is provided in a minimally invasive way, potentially reducing hospitalization rates and relieving symptoms [29,31]. However, it is essential to consider the associated risks. We revealed an incidence of major bleeding in the TTVI group. Chick et al. [32] reported that TTVI resulted in 7.4 % bleeding complications, further supporting our findings. This increased bleeding risk, primarily observed within the first month after TTVI, is likely potentiated by renal insufficiency, bleeding history, and periprocedural combination anticoagulation and antiplatelet therapy. However, it must be taken into account that these were the results of only two studies [12,13].

These findings demonstrated the potential role of TTVI as a treatment option for patients with severe TR who are deemed as high surgical risk. Future investigations should aim at determining the population that derives the most benefit from TTVI, optimizing procedural approaches to limit adverse events, and assessing whether a durable response to treatment is achievable. Long-term follow-up will be important to assess whether the observed quality-of-life improvements translate into sustained clinical benefits. Further studies about TTVI with longer-term data, including mortality and HF hospitalization, using prolonged follow-up RCTs are needed to elucidate any sustained benefit. Patient selection is essential for optimizing outcomes following TTVI. Based on current evidence, patients with atrial functional tricuspid regurgitation, preserved right ventricular function, NYHA functional class II to III symptoms, and PASP < 70 mmHg are the ones most likely to derive the greatest symptomatic and hemodynamic benefits [33], comprehensive clinical and echocardiographic evaluation within a multidisciplinary Heart Team framework is needed to identify the most appropriate candidates for interventions tailored to provide maximal benefit and minimal procedural risk.

Prior *meta*-analyses demonstrated statistically significant improvements in cardiac function and echocardiographic parameters following transcatheter repair [34,35]. However, these analyses were based on single-arm observational studies, limited comparisons to pre- and post-repair status within the same patient cohort, increasing the potential for bias due to the lack of a control arm. Furthermore, the analysis by Fu et al. included mainly observational studies and incorporated only one RCT [36]. Compared to these prior analyses, our analysis represents the most comprehensive and updated analysis on this topic. We also included three RCTs and only PSM observational studies to minimize selection bias, and included the recently published 2-year follow-up data from the TRILUMINATE trial. In addition, we integrated IPD reconstructed from Kaplan-Meier curves, enhancing survival estimates through restricted mean survival time modeling.

This analysis should be viewed in the context of certain limitations. There were differences between the studies in terms of the design and patient selection criteria. Notably, the participating patients were typically treated with beta-blockers and diuretics, while more recent therapies, such as angiotensin receptor-neprilysin inhibitors (ARNI) and sodium-glucose cotransporter-2 inhibitors (SGLT2i), were nearly absent. Nonetheless, conventional medical therapy has not shown benefit among patients with severe TR. Moreover, the majority of RCTs data were conducted in North America and Europe. The analysis included early-generation devices, whereas newer systems may have improved safety profiles. The inclusion of observational studies might have introduced selection and ascertainment biases; however, we restricted our inclusion to PSM analyses and performed a subgroup analysis based on the study type. There was evidence of moderate to substantial statistical heterogeneity on the outcomes of all-cause mortality and HF hospitalization, which was mitigated in the subgroup analysis according to the study type.

## 5 Conclusion

In this *meta*-analysis of 5 studies, TTVI did not significantly reduce all-cause mortality. TTVI was associated with reduced TR severity and improved quality of life, but was associated with a higher incidence of major bleeding. TTVI was also associated with a reduction in HF hospitalization, a finding which was driven predominantly by non-randomized data and should be interpreted with caution. TTVI might offer a therapeutic option for inoperable patients with symptomatic severe TR. Future large RCTs are needed to confirm these findings and identify the subset of patients who benefit the most.

## Ethical approval

Not applicable.

## CRediT authorship contribution statement

**Ahmed Ibrahim:** Writing – original draft, Investigation, Conceptualization. **Laila Shalabi:** Methodology, Investigation, Conceptualization. **Sofian Zreigh:** Formal analysis. **Shrouk Ramadan:** Writing – original draft, Project administration. **Ahmed Samir:** Methodology, Data curation. **Mohamed Adel Elsawy:** Methodology, Investigation. **Mohamed Mahmoud Fathy:** Methodology, Investigation. **Belal Mohamed Hamed:** Methodology, Investigation. **Hossam Elbenawi:** Methodology, Investigation. **Mustafa Turkmani:** Writing – review & editing, Supervision. **Ahmed Y. Azzam:** Supervision, Validation. **Hani Mahmoud-Elsayed:** Supervision. **Islam Y. Elgendy:** Writing – review & editing, Supervision.

## Funding

None.

## Declaration of competing interest

The authors declare the following financial interests/personal relationships which may be considered as potential competing interests: Ahmed Y. Azzam reports article publishing charges was provided by Seoul National University (SNU). If there are other authors, they declare that they have no known competing financial interests or personal relationships that could have appeared to influence the work reported in this paper.
