# Supplementary material for: Transcatheter tricuspid valve intervention versus optimal medical therapy alone for severe tricuspid regurgitation: an updated meta-analysis with reconstructed time-to-event data
Source: Int J Cardiol Heart Vasc. 2025 Sep 13;61:101794. doi: 10.1016/j.ijcha.2025.101794 (PMC12744525; doi:10.1016/j.ijcha.2025.101794)
Supplement: Supplementary Data 1 [file mmc1.docx]

Supplementary materials

**Figure S1:** Risk of Bias of included RCTs.

**Figure S2:** Risk of Bias of included observational studies. **Figure S3:**  Kaplan-Meier survival analysis for all-cause mortality_RCTs subgroup.
**Figure S4:**  Kaplan-Meier survival analysis for all-cause mortality _observational studies subgroup.

**Figure S5:** Forest Plot of Heart failure-specific mortality.
**Figure S6:** Forest Plot of Stroke.

**Table S1:** Search terms used in each database.


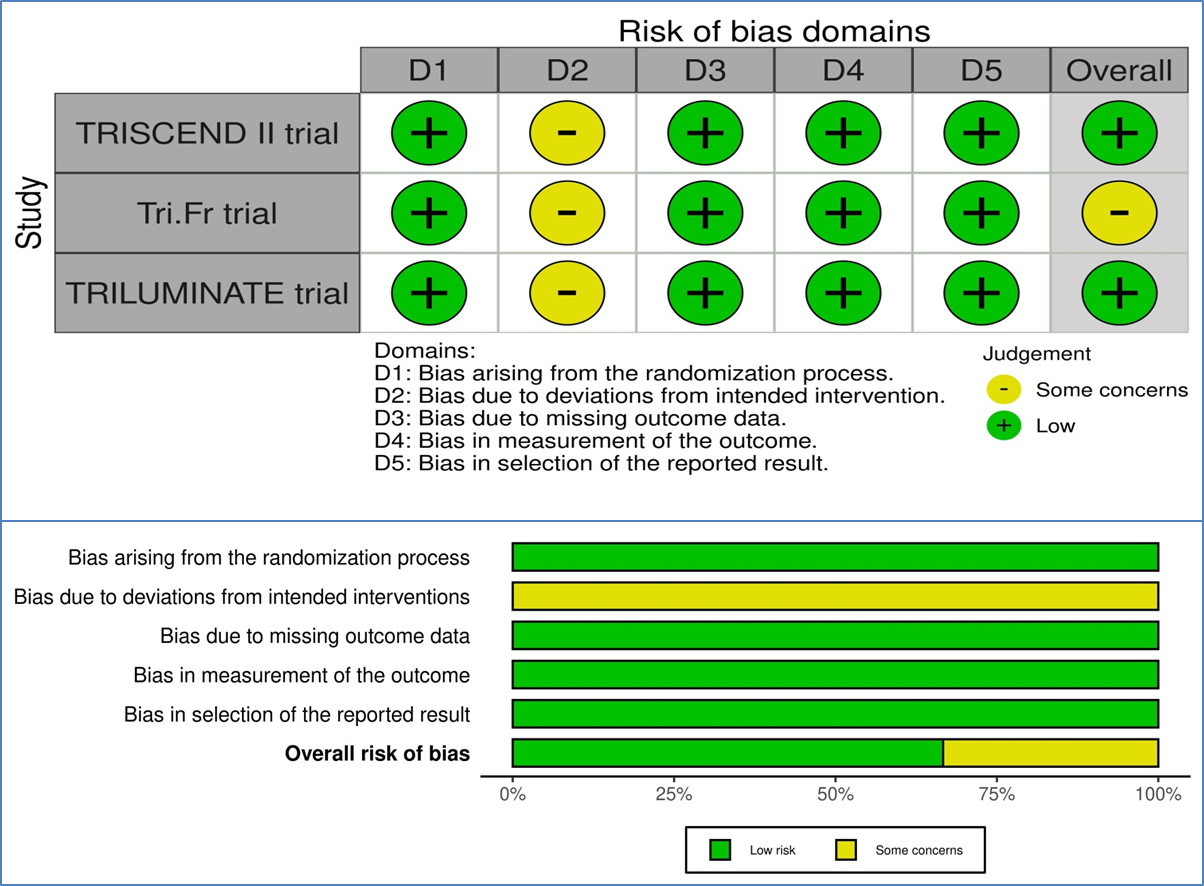
**Figure S1: Risk of Bias of included RCTs.**


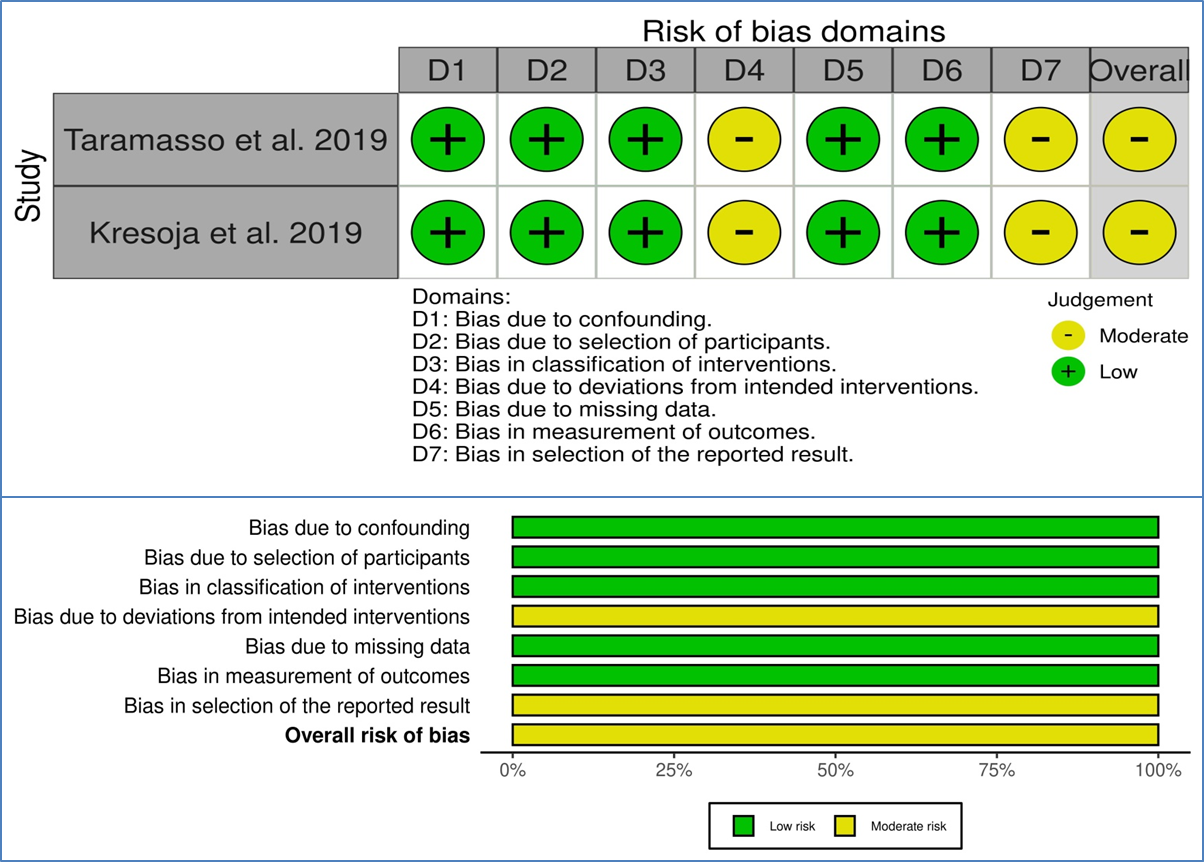
Figure S2: Risk of Bias of included observational studies.

**
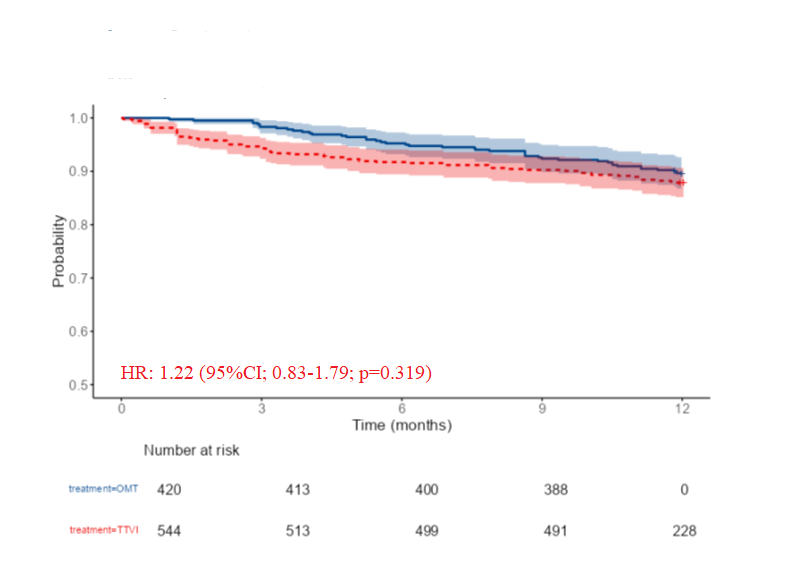
Figure S3:  Kaplan-Meier survival analysis for all-cause mortality_RCTs subgroup.**


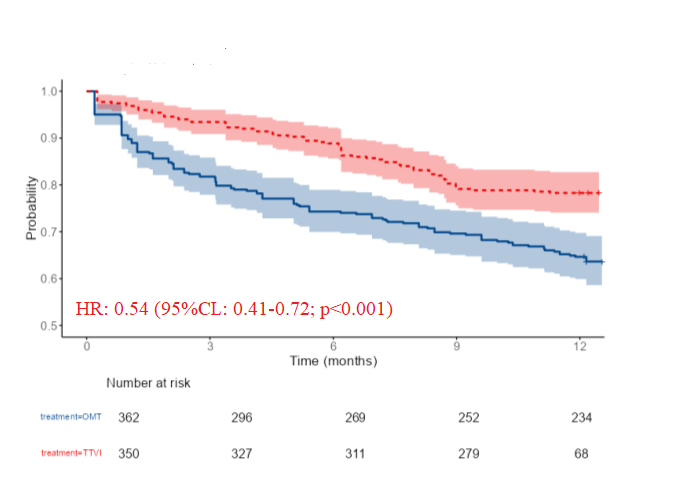
 **Figure S4:**  **Kaplan-Meier survival analysis for all-cause mortality _observational studies.**

**
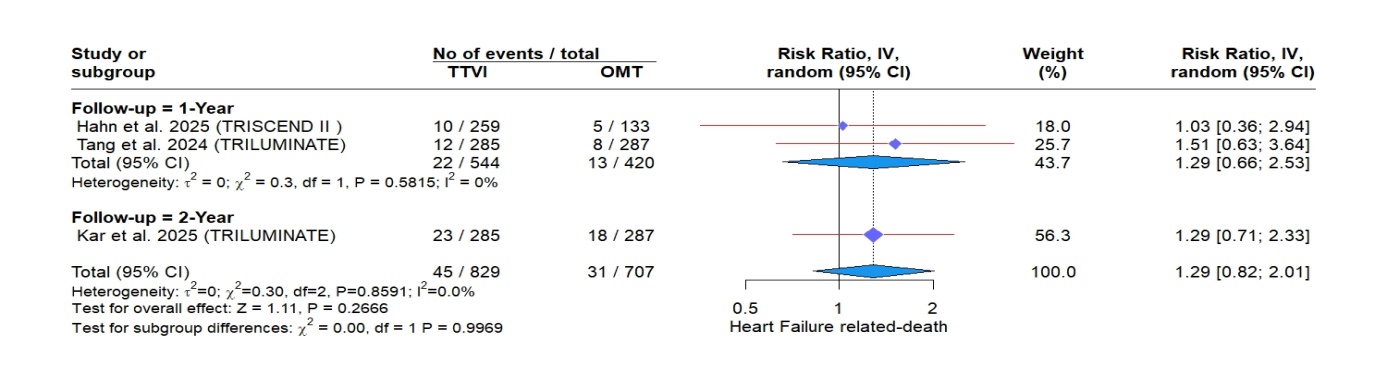
Figure S5: Forest Plot of Heart failure-related mortality.**

**Figure S6: Forest Plot of Stroke.**


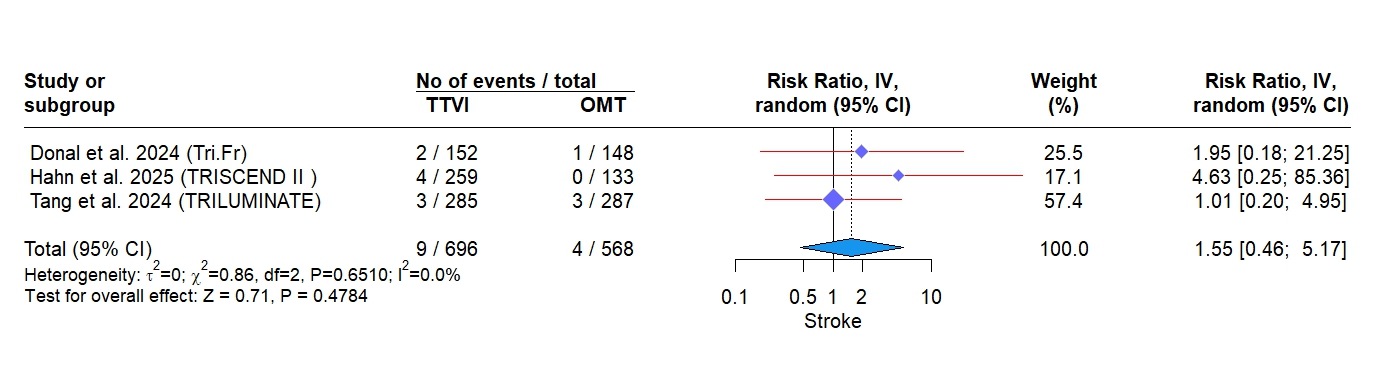


**Table S1: Search terms used in each database.**

| Database | Term | Number |
| --- | --- | --- |
| PubMed | (Transcatheter[Title/Abstract] OR Percutaneous[Title/Abstract] OR Catheterization[Title/Abstract]) AND (Intervention[Title/Abstract] OR Repair[Title/Abstract] OR Replace*[Title/Abstract] OR Implant*[Title/Abstract]) AND ("Medical Therapy"[Title/Abstract] OR "Drug Therapy"[Title/Abstract] OR "Conservative Treatment"[Title/Abstract]) AND ("Tricuspid Valve Regurgitation"[Title/Abstract] OR "Tricuspid Valve Insufficiency"[Title/Abstract] OR "Tricuspid Regurgitation"[Title/Abstract] OR "Tricuspid Insufficiency"[Title/Abstract]) | 100 |
| Web of Science | TS= TS= ((Transcatheter OR Percutaneous OR Catheterization) AND (Intervention OR Repair OR Replace* OR Implant*) AND ("Medical Therapy" OR "Drug Therapy" OR "Conservative Treatment") AND ("Tricuspid Valve Regurgitation" OR "Tricuspid Valve Insufficiency" OR "Tricuspid Regurgitation" OR "Tricuspid Insufficiency")) and Proceeding Paper or Letter or Early Access or Editorial Material or Review Article (Exclude – Document Types) ((Transcatheter OR | 67 |
| SCOPUS | TITLE-ABS-KEY ( ( transcatheter OR percutaneous OR catheterization ) AND ( intervention OR repair OR replace* OR implant* ) AND ( "Medical Therapy" OR "Drug Therapy" OR "Conservative Treatment" ) AND ( "Tricuspid Valve Regurgitation" OR "Tricuspid Valve Insufficiency" OR "Tricuspid Regurgitation" OR "Tricuspid Insufficiency" ) ) AND ( EXCLUDE ( DOCTYPE , "re" ) OR EXCLUDE ( DOCTYPE , "ed" ) OR EXCLUDE ( DOCTYPE , "le" ) OR EXCLUDE ( DOCTYPE , "ch" ) OR EXCLUDE ( DOCTYPE , "no" ) OR EXCLUDE ( DOCTYPE , "sh" ) OR EXCLUDE ( DOCTYPE , "er" ) OR EXCLUDE ( DOCTYPE , "cp" ) ) | 188 |
| COCHRANE | (transcatheter OR percutaneous OR catheterization) AND (intervention OR repair OR replace* OR implant*) AND (“Medical Therapy" OR "Drug Therapy" OR "Conservative Treatment”) AND (“Tricuspid Valve Regurgitation" OR "Tricuspid Valve Insufficiency" OR "Tricuspid Regurgitation" OR "Tricuspid Insufficiency" ) in Title Abstract Keyword - (Word variations have been searched) | 39 |
| Total |  | 390 |
